# Leaders’ creation of shared identity impacts group members’ effort and performance: Evidence from an exercise task

**DOI:** 10.1371/journal.pone.0218984

**Published:** 2019-07-11

**Authors:** Mark Stevens, Tim Rees, Niklas K. Steffens, S. Alexander Haslam, Pete Coffee, Remco Polman

**Affiliations:** 1 Bournemouth University, Department of Sport and Physical Activity, Faculty of Management, Poole, United Kingdom; 2 School of Psychology, The University of Queensland, Brisbane, Australia; 3 Faculty of Health Sciences and Sport, University of Stirling, Stirling, United Kingdom; 4 School of Exercise and Nutrition Sciences, Queensland University of Technology, Brisbane, Australia; University of Auckland, NEW ZEALAND

## Abstract

There is growing evidence that leaders’ effectiveness derives in part from their creation of a sense of identity that is shared by members of a group they are attempting to lead (i.e., their identity entrepreneurship). Little is known, however, about the impact of identity entrepreneurship in sport and exercise settings, particularly in relation to its effect on group members’ effort and performance. Using a pre-post between subjects experimental design, we examined the effect of leaders’ identity entrepreneurship on group members’ effort and performance during 5km cycling time trials. Following a baseline session (in which time trials were completed individually), participants (*N* = 72) were randomly allocated to either a high or low identity entrepreneurship condition, and further randomly divided into groups of five (including a leader who was a confederate). In the subsequent test sessions (which participants attended with their fellow group members), leaders displayed either high or low identity entrepreneurship behaviors. Results indicated that, compared to participants in the low identity entrepreneurship condition, those in the high identity entrepreneurship condition maintained greater effort (maximum heart rate), and demonstrated improved (rather than poorer) performance (average power output in the first 60 seconds of time trials). Examination of pacing showed that the largest increases in participants’ average power output occurred in the early stages of their second time trials for those in the high identity entrepreneurship condition only. Results provide causal evidence that leaders who create a shared sense of identity among team members are able to inspire greater participant effort and performance.

## Introduction

A key indicator of any leader’s effectiveness is their ability to elicit maximum effort and, ultimately, performance from those they lead. In sports settings, for example, this ability can be crucial for a manager attempting to guide his or her team to a league championship or to safety from relegation. Indeed, evidence suggests that formal and informal leaders of sports teams can influence various outcomes including team confidence [1, 2], team cohesion [3], team member motivation [4], and team and individual performance [5, 6], and that fitness class leaders can influence (both positively and negatively) group members’ affective exercise experiences [7, 8] and intentions to remain in the class [9].

Nevertheless, *how* leaders can most effectively foster positive group member behaviors and outcomes (including greater effort and performance) remains a (possibly *the*) key question for leadership researchers. Here, the body of literature pertaining to leadership effectiveness is considerable and spans a wide array of contexts (i.e., not just sport and exercise, but also, business, educational, and military; e.g., see [10–12] for reviews). This research has shed light on a diverse array of determinants of leadership effectiveness (and perceptions thereof). For example, meta-analytic evidence points to (1) the salient impact of gender on perceptions of leader effectiveness [13], (2) the value of multiple group members taking on leadership roles (i.e., through shared leadership; [14]), and (3) the value of ‘transformational’ leadership behaviors [15]. While a major contribution of research based on the transformational leadership approach [16, 17] has been to draw attention to the importance of leader-follower relationships, a large body of recent research underpinned by the *social identity approach to leadership* has indicated the centrality of group processes to leadership effectiveness (see [18] for a review). That is, in contrast to traditional leadership approaches that concentrated on the traits and extraordinary abilities of ‘great men’ (with limited success; e.g., see [19, 20]), this research has pointed to the role that a sense of shared group membership plays in allowing leaders to exert influence over a group [18, 21, 22]. In particular, a body of recent research indicates that the extent to which leaders act as *entrepreneurs of identity* by actively cultivating a sense of ‘we’ and ‘us’ among group members [23, 24] has positive implications for various outcomes including group members’ engagement, burnout, and turnover intentions [25, 26] and their support for, and endorsement of, the leader [27, 28].

Building on this promising research, in the present study, we explore the possibility that leaders’ engagement in identity entrepreneurship may directly influence group members’ effort during a task, and therefore that, all else being equal, identity entrepreneurship is a precursor to improved task performance. In this way, we also seek to build on previous research indicating the salient role that other group processes (e.g., cohesion) can play in influencing group members’ task-related effort and performance [29–31]. Specifically, we test these ideas in the context of a physical task (a 5km cycling time trial) to shed light on the impact of leaders’ identity entrepreneurship in sport and exercise settings—settings in which it has received minimal attention to date.

### Leaders’ promote positive group and individual outcomes by creating social identity

The social identity approach [32, 33] is built on the assertion that individuals can categorize themselves, and behave, both in terms of personal identity (i.e., as ‘I’ and ‘me’) and social identity (i.e., as ‘we’ and ‘us’). The approach further suggests that when people categorize themselves, and others, in terms of a shared social identity (e.g., as a member of a particular exercise group or sports team) this lays the foundation for various group behaviors including social collaboration [34], social influence [35], and, of particular relevance to the present article, leadership [18, 36, 37]. According to social identity theorizing, effective leadership is therefore considered contingent on the leader’s capacity to create, represent, advance, and embed a shared sense of identity among group members [18, 24].

Supporting this assertion, empirical evidence suggests that the benefits associated with identity leadership include increases in group members’ satisfaction [38–40] and support for leaders [41, 42], as well reductions in their turnover intentions [26, 38, 40] and experience of burnout [25, 26]. Notably too, in line with social identity theorizing [18], identity leadership has also been associated with greater social identification [6], with additional experimental research demonstrating, in turn, that this increased social identification is related to greater productivity [43], commitment [44], and effort [45].

Previous research in the social identity tradition has also shown that leader effectiveness is contingent on leaders’ ingroup prototypicality—that is, the extent to which they are perceived to embody the norms, beliefs, and values of a salient social identity [18, 22, 46]. As yet, though, we know little about the extent to which leader effectiveness is *causally* impacted by leaders’ creation of shared identity through acts of identity entrepreneurship—a question that is particularly relevant in the case of new groups. This is because research concerning identity entrepreneurship has often used retrospective designs—in particular, to explore leaders’ use of language (i.e., building on the notion that *collective* language is one way through which leaders may demonstrate their identity entrepreneurship; [18]). Steffens and Haslam [27] showed that victorious candidates in Australian Prime Ministerial elections between 1901 and 2010 made 61% more references to ‘we’ and ‘us’ and used these words with greater regularity than unsuccessful candidates (once every 79 words versus once every 136 words). Similarly, following an analysis of media data (i.e., interviews, speeches, team announcements, and blog posts) emanating from six prominent leaders at the 2012 Olympic Games (four performance directors, the leader of Team GB, and the chairman of the Games organizing committee), Slater et al. [47] observed that less successful leaders (including one who left his position following the Games) tended to refer to their team members as ‘they’ rather than ‘we’ or ‘us’.

Only recently has the first attempt been made to examine the effects of identity entrepreneurship using a more rigorous (i.e., two-wave) design. Steffens et al. [26] administered two surveys (separated by a 10-month interval) to examine the effect of identity entrepreneurship on manual workers’ subsequent burnout, work engagement, and turnover intentions. In line with predictions, results showed that workers’ perceptions of their leaders’ identity entrepreneurship at Time 1 predicted greater work engagement and lower burnout and turnover intentions at Time 2 (while there was no evidence of the reverse path). But while these results provide some evidence of directionality, the longitudinal design is not able to rule out the possibility of alternative explanations—something that can only achieved through experimental research.

In sport and exercise contexts, no attempt has been made to test the effects of identity entrepreneurship either longitudinally or experimentally. However, reflecting the growing influence of social identity theorizing in these domains, there has been a recent proliferation of efforts to test the impact of leaders engaging in identity leadership more broadly, with research suggesting that this has a range of benefits [6, 48, 49]. Of particular relevance is a recent experimental study by Fransen et al. [6] that used two interactive soccer tasks (involving passing as well as dribbling and shooting). This found that group task performance was influenced by the confidence a confederate leader expressed in their teams. Crucially, Fransen et al. [6] also found that participants’ (1) perceptions of confederate leaders’ engagement in identity leadership, and (2) identification as a member of their newly formed team, mediated the relationship between the perceived confidence the leader showed in the team and participants’ perceptions of their individual performance on the two soccer tasks. But while these findings indicate that identity leadership is positively related to group members’ performance, there remains a clear need for research examining causal relationships between leader identity entrepreneurship and group members’ behaviors in sport and exercise settings (in particular, those related to their effort and performance).

### The present research

The present research sought to address these lacunae. More specifically, our study extended previous research in at least three key ways. First, rather than assessing the mediating effect of identity leadership [6] we manipulated this (or, more specifically, leader identity entrepreneurship) in order to examine the extent to which this has a causal impact on participants’ behavior. To achieve this, participants completed a baseline test before we randomly allocated them to either a condition where a confederate leader displayed high identity entrepreneurship or a condition where a confederate leader displayed low identity entrepreneurship. Extending previous research on identity entrepreneurship [25, 26] and identity leadership in sport more broadly [6], this allowed us to assess the causal effects of identity entrepreneurship on participants’ effort and performance. Second, by using a simple individual exercise task—a 5km cycling time trial on a static cycling ergometer—we were able to examine the relationship between identity leadership and objectively-assessed group member effort and performance. This builds on previous evidence of a positive association between identity leadership and group members’ perceptions of their own performance (i.e., a subjective performance measure; [6]), and on qualitative research indicating a positive relationship between identity leadership and sporting performance [47]. Third, by using this simple individual task (rather than, for example, an interactive skill-based task, such as those used by [6]) we were able to remove (or at least minimize) the impact of factors on participants’ performance that were unrelated to their effort (e.g., participants’ technique, concentration, and co-ordination with other team members). As such, we were able to detect whether any performance improvements observed resulted from increases in group members’ efforts.

In line with social identity reasoning, our hypotheses were as follows:

**H1.** Leaders’ identity entrepreneurship will have a positive impact on group members’ efforts during a physical exercise task.
**H2.** Leaders’ identity entrepreneurship will have a positive impact on group members’ performance of a physical exercise task.

## Methods

### Participants

We recruited a sample of 88 recreationally active participants from a British University (the first author’s institution). Specifically, an opportunistic sampling strategy was used whereby individuals who (1) specified that they had no health conditions that inhibit their day-to-day engagement in physical activity, and (2) stated that they were physically capable of completing the 5km time trial task on two occasions were recruited through word of mouth and advertisements to the student body (via email and social media). Four participants withdrew between the two test sessions. As a result, in the second phase of the experiment—which involved a group-based manipulation (see below)—group size (excluding the confederate) was reduced from four to three participants in four instances. In order to rule out effects due to varying group size and to guarantee comparability of participant behavior across groups (described in more detail below), these groups were excluded, leaving a final sample of 72 participants. Characteristics of the final sample are presented in Table 1. Height and weight were measured using a standard stadiometer (Seca 213, Germany) and scale (Seca 807, Germany).

**Table 1:** Participant anthropometric data.

	**Male** **(***n* **= 40)**	**Female (***n* **= 32)**	**High identity entrepreneurship condition (***n* **= 36)**	**Low identity entrepreneurship condition (***n* **= 36)**	**Total** **(***n* **= 72)**
Age (years)	22.30±1.83 (19–31)	21.22±1.74 (18–24)	22.11±2.15 (18–31)	21.53±1.48 (18–24)	21.82±1.86 (18–31)
Height (cm)	180.36±7.51 (166.50–194.00)	170.57±7.14 (155.50–188.00)	176.49±9.34 (163.00–194.00)	174.53±8.06 (155.50–189.00)	176.01±8.79 (155.50–194.00)
Weight (kg)	79.39±10.94 (63.00–120.40)	71.64±11.07 (50.00–97.90)	77.78±13.42 (50.00–120.40)	74.11±9.23 (52.20–91.60)	75.94±11.58 (50.00–120.40)

Notes: Data are presented as Mean ± SD (range); SD = standard deviation; the high and low identity entrepreneurship conditions both comprised 20 males and 16 females; all data were collected immediately prior to time trial 1.

Before participating in the study, all participants completed a health questionnaire to identify any contraindications to strenuous exercise. Participants were told that the study’s aim was to examine time trial performance under different conditions, and informed that participation would involve completing two 5km time trials on separate days. All participants gave written consent based on this information. Ethical approval for the study procedures was obtained from Bournemouth University Research Ethics Committee on 31^st^ January 2017 (project reference ID 14123).

### Power analysis

Given the novel paradigm and design of the present experiment, we could locate just two studies (with four effect sizes) to provide guidance regarding necessary sample size (and thus our sample size estimates should be considered vague approximations). The first (a small study; *N* = 18) examined the effect of a manipulation to enhance participants’ identification as members of a newly formed team on subsequent performance during one- and three-minute cycling time trials, reporting effect sizes equivalent to Cohen’s *d*s of 1.19 and 2.11 [45]; the second (*N* = 80 across two experiments) examined the impact (within a dart-throwing task) of failure feedback delivered by an in-group versus an out-group experimenter on subsequent performance, reporting effect sizes of *d* = 0.89 and *d* = 1.22 [50]. Using the lowest effect size estimate (*d* = 0.89) with an alpha of .05, power of .80, and a one-tailed effect, sample size estimates (G*Power; [51]) indicated that *N* = 34 would be required. Recognizing this small sample size, and given recent concerns about small sample size research (and related issues of reproducibility and replicability; e.g., see [52, 53]), we aimed for a minimum sample of 64—that is, sixteen groups of four with an equal split across conditions of male and female groups. To protect against participant dropout, however, we tested in excess of this minimum sample amount at baseline (i.e., a total of *N* = 88). As a result of the withdrawal of four participants between sessions, 12 of the original sample conducted Trial 2 in groups of three (i.e., four groups had one participant drop out prior to the Trial 2 sessions; thus, there were four groups containing just three participants each); by subsequently excluding those participants who conducted Trial 2 in groups of three (see preliminary analysis section below for the reasons behind this decision), we ended with an effective sample size of *N* = 72, sufficient to detect effect sizes of *d* = 0.60.

### Experimental procedures

Participants visited the laboratory on two occasions separated by a period of one to two weeks. On both occasions, participants completed a five-minute self-paced warm up followed by a 5km time trial on a cycling ergometer (Wattbike Pro, Nottingham, UK). Resistance levels were standardized for all time trials, with the air break set to 5 and the magnetic brake to 1 (mirroring flat cycling conditions). To overcome flywheel inertia, participants were asked to begin cycling at a self-selected comfortable cadence immediately prior to beginning both time trials. During the time trials, participants were blind to all information except distance remaining. No verbal encouragement was given but participants were permitted to drink water *ad libitum*. Participants were asked to refrain from (1) high intensity exercise in the two days prior to the testing sessions, and (2) consuming caffeine in the two hours prior to the testing sessions. Participants’ testing sessions were conducted at the same time of day to minimize the influence of circadian variance [54], and at an ambient temperature of 21°C.

Prior to Trial 1, participants were given time to familiarize themselves with the cycling ergometer and adjust the saddle and handlebar positions to their liking. These positions were recorded and replicated for participants’ second time trials. Participants attended the laboratory individually on their first visit and completed their time trial with only the first author or a research assistant present. The primary instruction given was to complete the test ‘as fast as you can’.

Before the second round of testing, participants were allocated (via random number generation) to same-gender groups of four. For their second visit, participants were asked to attend the laboratory at the same time as their fellow group members. Once all group members had arrived, the experimenter (the first author) collectively informed participants that (1) a team competition had been organized, (2) one person had been chosen at random from each group to be the ‘team leader’, (3) this person had been contacted in advance to confirm they were happy to fulfil this role, and (4) this person had been provided some additional information about the competition. The experimenter then asked the team leader to relay this information to the group while the experiment set up was finalized and left the room. In all instances, the individual identified as the leader was a confederate who, as far as participants were aware, was a fifth group member. At this stage the confederate informed the group that there was “a competition that involves 12 groups of students and the results for the total time taken by each group will be sent round afterwards by email as a leaderboard”, before delivering the manipulation (see below). Following this, participants were called in turn into a separate room to complete their second time trials, with the confederate called last in all instances. When each participant had finished their time trial, they left the laboratory.

### Manipulation

The gender of the confederate was matched to the gender of the group members to prevent this acting as a confounding variable. A single male confederate was used for all male groups, while two female confederates were used with each completing four of the eight female groups (two high identity entrepreneurship and two low identity entrepreneurship groups each) included in our final sample.

In developing manipulation scripts, we drew on previous identity entrepreneurship and social identity research. First, we drew on evidence linking leaders’ effectiveness to their greater use of collective (rather than personal) pronouns; that is, evidence that we-referencing language—a strategy through which leaders can demonstrate their identity entrepreneurship [18]—can help engender support from, and mobilize, followers [27]. Several references to ‘we’ were therefore included in the high identity entrepreneurship manipulation, while several references to ‘I’ were included in the low identity entrepreneurship manipulation. Second, we included a strategy that has previously been used to build individuals’ identification as a member of a newly formed team—the creation of a team name [45]. By asking the confederate leaders to suggest this in the high (but not the low) identity entrepreneurship condition, we provided the leaders with a tool to demonstrate their desire to create a sense of ‘us’. Finally, the content (i.e., underpinning message) of the high and low identity entrepreneurship manipulations aimed to emphasize (and reinforce throughout the session) the importance (or not in the low identity entrepreneurship condition) of the team.

Specifically, the manipulation delivered by confederates in the high identity entrepreneurship condition was: “So we’re all in this together basically. Let’s give this a really good go as a team. If we all do our best then we can do well here. We want to try and win don’t we? Maybe we should come up with a team name. Any ideas?” Confederates were also provided with a list of phrases to use between one participant coming out of, and the next going into, the testing room: “Great stuff (person’s name) you look exhausted, looks like you’ve really given your best for the team”, “Well done (person’s name) another big effort for team X”, “Come on (person’s name) do it for Team X”. Confederates used one phrase in the transition between each of the other group members’ time trials.

For the low identity entrepreneurship condition, the confederate followed their explanation of the competition by delivering the following manipulation: “I wouldn’t worry too much about that though personally. It’s not really a group thing, it’s an individual task and what everyone does is up to them. I wouldn’t worry about what anyone else is doing, just do your own thing.” In this condition confederates were instructed to say nothing in the transition time between participants’ time trials.

### Debrief

Following the experiment, participants were informed of the study’s aims and procedure and provided with a general summary of the results (all via email). Upon request, participants were also provided with their individual results. No leaderboard pertaining to group performance was circulated and no participants were given details of any other participant’s individual results.

### Measures

#### Manipulation check

Following their second time trial (and while still in the testing room separate from their fellow participants and ‘team leader’), participants completed the four entrepreneurship items of the Identity Leadership Inventory [24] (e.g., “This leader made people feel as if they are part of the same group”). Items were scored on scales ranging from 1 (not at all) to 7 (completely). The four items were summed and divided by four to obtain a mean score for each participant. In line with previous research [25], this measure demonstrated good internal consistency (Cronbach’s α = .89), while confirmatory factor analyses (using AMOS 23.0; [55]) supported the psychometric properties of the scale: χ2[2] = 6.03, *p* = .049; CFI = .98; SRMR = .03; RMSEA = .17; PCLOSE = .08.

#### Effort

Given the linear relation between intensity of work and heart rate (HR) [56], HR represents an appropriate physiological index of effort (e.g., see [57]). As such, participants’ average and maximum HR (measured in beats per min; Polar H7, Finland) were used as indicators of effort during the time trials. Although, as expected, these measures were highly correlated (Trial 1, *r* = .91, Trial 2, *r* = .84, both *ps* < .01), both were included in the final index of effort because they offer slightly different measures of effort. That is, average HR provides an indication of effort exerted across the whole time trial, while maximum HR indicates participants’ maximum effort exerted.

#### Performance

Three objective indicators of performance were obtained during time trials: time taken (seconds), average power output, and average power output over the first 60 seconds (both measured in watts). We analysed the first 60 seconds in addition to whole time trials because of the primacy of this period to the manipulation (i.e., either the initial statement, or, in the high identity entrepreneurship condition, a phrase delivered by the confederate to reinforce it), and research indicating that identity-based manipulations elicit immediate performance effects [45, 50]. Nevertheless, to gain a broader understanding of the development of performance over the duration of the time trials, we also examined participants’ average power output over each 250m interval, allowing a visual inspection of variations in participants’ pacing across the two time trials.

## Results

### Preliminary analysis

Data were screened for missing values, outliers, and indices of non-normality. For four participants’ first time trials, raw data files revealed either partial or complete missing data for HR (due to equipment failing to detect HR throughout time trials). Values for average and maximum HR were treated as missing for these participants, and listwise deletion was used to handle these missing data in subsequent analyses. No other missing values were observed. For the purposes of detecting outliers (and subsequent analyses), we calculated gain scores by subtracting participants’ Trial 1 results from their Trial 2 results for each variable (i.e., each indicator of effort and performance). Given evidence that outlier removal has substantial benefits for error rates in *t*-tests and ANOVAs [58], we then identified, and removed, gain score outliers within each condition (high and low identity entrepreneurship) for each variable using the Median Absolute Deviation (MAD) equation with a cut-off of 2.5 (i.e., the median ±2.5 times the MAD; [59]). This approach is a more robust measure of dispersion than mean ± 2 or 3 standard deviations [59]. Following the removal of outliers, Shapiro-Wilk Tests indicated that gain scores for all indicators of effort and performance followed a normal distribution across both conditions.

Next, given the dropout of four participants, and the potential for different group sizes to influence group dynamics (and therefore our results; e.g., see [60]), we tested for the effect of group size. To do this, we conducted one-way analyses of variance (ANOVAs) on the gain scores for each dependent variable (i.e., equivalent tests to the independent *t*-tests used throughout our main analyses, see below) and entered group size as an additional fixed factor. Results revealed a significant condition (i.e., manipulation) X group size interaction for average HR (*p* = .017). Given this significant interaction, previous evidence that group size can influence members’ effort (and therefore their team's performance; e.g., [61, 62]), and to enhance reliability, we refrained from including participants who completed the second session in groups of three in subsequent analyses (but return to the results of analyses in which these participants were included in the Limitations and Future Research section). At this stage, gain score outliers were therefore recalculated, and removed, for each dependent variable (using the same MAD criteria) for the final sample of 72. Of these 72 participants, only three had missing data for HR and, following the removal of outliers, Shapiro-Wilk Tests once again indicated that gain scores for all dependent variables followed a normal distribution in both conditions. Further analyses (using independent *t*-tests) revealed no significant differences in pre-test scores between participants in the high and low identity entrepreneurship conditions.

### Main analyses

#### Analytic approach

Analyses based on gain scores were considered more appropriate than analyses of covariance (ANCOVAs) with pre- and post-test scores entered as covariates and dependent variables respectively. This is because our primary interest was in how the two conditions, on average, differed in gains, rather than how participants differed at post-test, given that they started with the same score—the question tested by ANCOVAs [63]. Indeed, with the exception of (some) randomized control trials, several researchers recommend against using ANCOVAs in pre- and post-test designs [63–65]. We therefore conducted a series of independent *t*-tests to compare the gain scores of participants in the high and low identity entrepreneurship conditions. The assumption of equal variances (i.e., Levene’s test) was met for all dependent variables, and the Student’s *t*-test was used in all instances. The Bonferroni correction was applied separately to effort and performance outcomes (i.e., familywise). Alpha was therefore set at .025 for effort outcomes and .0167 for performance outcomes. In line with our directional hypotheses, one-tailed *p* values are reported for all indicators of effort and performance. Effect sizes are reported as Cohen’s *d*, with 0.2, 0.5, and 0.8 representing small, medium, and large effect sizes respectively [66]. Means and standard deviations for all dependent variables across the two conditions’ first and second time trials (following the removal of outliers) are presented in Table 2.

**Table 2:** Means and standard deviations for all dependent variables across the two conditions’ first and second time trials.

	**Time trial 1**		**Time trial 2**	
**Variable**	Mean	SD	Mean	SD
*High identity entrepreneurship condition*				
Average HR (beats per min)	167.57	10.42	165.67	11.14
Maximum HR (beats per min)	185.66	8.86	184.70	10.39
Time taken (seconds)	516.26	47.73	510.48	45.55
Average power output (watts)	178.24	38.97	181.18	39.15
Average power output first 60 seconds (watts)	167.23	57.95	181.70	51.13
*Low identity entrepreneurship condition*				
Average HR (beats per min)	168.09	13.84	162.88	13.69
Maximum HR (beats per min)	186.33	11.03	182.40	10.76
Time taken (seconds)	517.33	43.76	516.84	41.40
Average power output (watts)	173.91	41.56	174.12	40.49
Average power output first 60 seconds (watts)	193.93	63.56	182.39	54.52

Note: SD = standard deviation

Prior to conducting our main analyses, we tested for potential interactions between our manipulation and (1) participants’ gender, and (2) the confederate participants were exposed to in our manipulation. To do this, we conducted two additional sets of one-way ANOVAs on the gain scores for each of our dependent variables with gender and confederate separately entered as additional fixed factors. We observed no significant condition X gender or condition X confederate interactions, and therefore report the results of analyses in which we do not control for these factors.

We note that, although our focus was examination of differences between the two conditions, our methodological approach—in which participants were nested within small experimental groups of four (plus a confederate)—meant that within-group dependencies were possible. To ascertain the magnitude of the dependencies present in our sample, we used maximum likelihood estimation to calculate intra-class correlations (ICCs) for each of our dependent variables in each condition separately. ICC values ranged from 0 to 0.38, with all values except one ≤0.24 (see Table 3). Notably, these analyses also indicated that the variance explained at the group level was not significantly different from zero in any of the models (i.e., *p*’s > .05 in all instances where at least some variance was explained at the group level). In addition, we examined changes (within the high and low identity entrepreneurship conditions) in -2 log likelihood values for models (1) without the group-level effect, and (2) with the group-level effect added. We then tested the significance of these changes relative to the change in degrees of freedom (i.e., 1)—a test possible because the -2 log likelihood statistic has a chi-square distribution [67]. These analyses showed that adding the group-level effect significantly improved fit in just 1 of the 10 models (see Table 3 for details of these analyses).

**Table 3:** Diagnostic statistics relating to group-level effects for each of our dependent variables across the two conditions.

Variable	Low identity entrepreneurship condition		High identity entrepreneurship condition	
	Intra-class correlation	-2 log likelihood change	Intra-class correlation	-2 log likelihood change
Average heart rate	0.24	2.33	0.00	0.00
Maximum heart rate	0.38	5.60*	0.09	0.23
Time taken	0.00	0.00	0.19	1.45
Average power output	0.00	0.00	0.20	1.66
Average power output first 60 seconds	0.05	0.10	0.14	0.71

Notes: -2 log likelihood change refers to differences in values between models in which the group-level effect was, and was not, included

*p < .05.

Although these analyses indicate that single-level analyses are appropriate in the present instance, and our focus remains on differences between conditions, our experimental design means that our data could be analysed using a multilevel framework. Thus, although our experiment does not meet the minimum thresholds to guarantee precise estimation of parameters, and avoid concerns regarding biased standard errors in a multilevel approach (i.e., we do not have a minimum of 50 groups and 30 people in each group; [68–70]), for descriptive purposes, and noting that our sample is substantially smaller than recommended for multilevel modelling, we provide details of multilevel analyses in the supplementary material (S1 Appendix). In short, these analyses demonstrated substantively similar results to those reported below (using single-level analyses) with regard to the impact of the experimental manipulation on our indicators of effort and performance.

#### Manipulation check

Supporting the efficacy of our manipulation, an independent *t*-test showed that confederates were perceived to engage in significantly more identity entrepreneurship in the high identity entrepreneurship condition (*M* = 5.05, *SD* = 1.22) than in the low identity entrepreneurship condition (*M* = 3.85, *SD* = 1.45), *t*(70) = 3.78, *p* < .001, *d* = 0.90.

**Tests of H1: Impact of leaders’ identity entrepreneurship on group members’ effort. Average HR:** Results showed that mean scores for average HR were lower for Trial 2 than Trial 1 in both the high (gain score mean = -1.90, *SD* = 7.29) and low (gain score mean = -5.55, *SD* = 9.46) identity entrepreneurship conditions. Further analyses showed that 46.7% of participants’ average HR scores were greater for Trial 2 than they were for Trial 1 in the high identity entrepreneurship condition, compared to 27.3% in the low identity entrepreneurship condition. With alpha set at .025 (as noted above), the two conditions’ gain scores were not significantly different from each other: *t*(61) = 1.701, *p* = .047, *d* = 0.43.

**Maximum HR:** Results showed that mean scores for maximum HR were lower for Trial 2 than Trial 1 in both the high (gain score mean = -.38, *SD* = 6.99) and low (gain score mean = -3.91, *SD* = 5.32) identity entrepreneurship conditions. Further analyses showed that 46.9% of participants’ scores for maximum HR were greater for Trial 2 than they were for Trial 1 in the high identity entrepreneurship condition, compared to 15.2% of participants in the low identity entrepreneurship condition. The difference between the two conditions’ gain scores was significant: *t*(63) = 2.30, *p* = .013, *d* = 0.57.

**Tests of H2: Impact of leaders’ identity entrepreneurship on group members’ performance. Time taken:** Results showed that participants were faster in Trial 2 than in Trial 1 in both the high (gain score mean = -5.78, *SD* = 24.74) and low (gain score mean = -.49, *SD* = 16.94) identity entrepreneurship conditions. Further analyses showed that 63.6% of participants’ Trial 2 times were faster than their Trial 1 times in the high identity entrepreneurship condition, compared to 40.6% of participants’ times in the low identity entrepreneurship condition. The difference between the two conditions’ gain scores was not significant: *t*(63) = -1.00, *p* = .160, *d* = .25.

**Average power output:** Results showed that participants produced greater average power output in Trial 2 than in Trial 1 in both the high (gain score mean = 2.94, *SD* = 22.97) and low (gain score mean = .21, *SD* = 17.25) identity entrepreneurship conditions. Further analyses showed that 61.8% of participants’ average power output scores were greater for Trial 2 than they were for Trial 1 in the high identity entrepreneurship condition, compared to 47.1% of participants in the low identity entrepreneurship condition. The two conditions’ gain scores were not significantly different from each other: *t*(66) = .56, *p* = .291, *d* = .13.

**Average power output first 60 seconds:** Results showed that, in the high identity entrepreneurship condition, participants produced greater average power output in the first 60 seconds in Trial 2 than in Trial 1 (gain score mean = 14.46, *SD* = 42.70). In the low identity entrepreneurship condition, participants produced lower average power output in the first 60 seconds in Trial 2 than in Trial 1 (gain score mean = -11.54, *SD* = 39.91). Further analyses showed that 64.7% of participants’ scores for average power output in the first 60 seconds were greater for Trial 2 than they were for Trial 1 in the high identity entrepreneurship condition, compared to 47.1% of participants in the low identity entrepreneurship condition. The difference between the two conditions’ gain scores was significant: *t*(66) = 2.59, *p* = .006, *d* = 0.63.

#### Further analyses of performance

**Fig 1:**
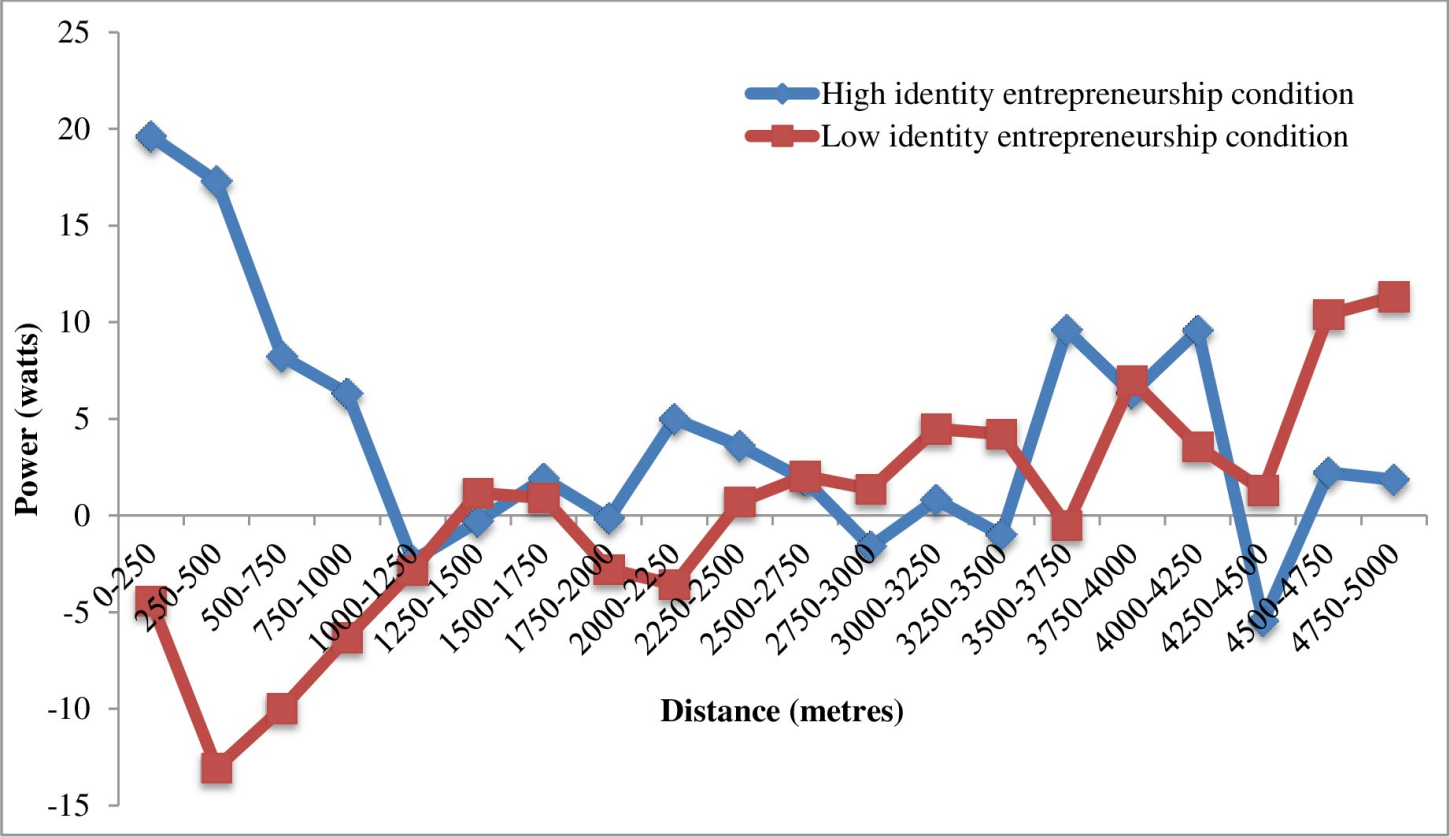
Mean difference in average power output between Trial 1 and Trial 2 for each 250m interval for the high and low identity entrepreneurship groups.

As noted above, to gain a greater understanding of performance effects, we conducted additional exploratory analyses examining participants’ pacing by averaging their power output data over 250m intervals. Having obtained this information, we first calculated gain scores for each interval (by again subtracting participants’ Trial 1 results from their Trial 2 results). Then, consistent with our previous analyses, we identified and removed gain score outliers in each condition (again using the MAD equation with a cut-off of 2.5; [59]). Based on this data, we plotted, and visually inspected, the mean difference in average power output for each 250m interval for the two conditions (see Fig 1).

Extending evidence of a significant difference between the two conditions’ gain scores over the first 60 seconds (see above), Fig 1 shows clear differences in the early stages (i.e., over approximately the first 1km) of participants’ time trials. While the low identity entrepreneurship condition’s average power output was lower in the early stages of Trial 2 than Trial 1, the high identity entrepreneurship condition’s power output was higher. Indeed, in the high identity entrepreneurship condition, the largest positive within condition difference of any stage of time trials is apparent in the first 500m, while the largest negative within condition difference is apparent during this period in the low identity entrepreneurship condition. Although the two condition’s difference scores then converge—such that changes for both conditions between Trial 1 and Trial 2 are similar between approximately the 1km and 4.5km points—visual inspection of Fig 1 also points to differences in the two condition’s gain scores at the end of the trial (i.e., the final ~ 500m). Here, the low identity entrepreneurship condition’s power output improved more from Trial 1 to Trial 2 than the high identity entrepreneurship condition’s power output. These differences were, however, smaller than those observed in the early stages of the time trials.

## Discussion

This study examined the impact of leaders’ identity entrepreneurship on group members’ effort during, and performance of, a physical exercise task. Hypotheses 1 and 2 were both partially supported, with results revealing significant differences—in favour of the high identity entrepreneurship condition—in gains from baseline to Trial 2 for maximum HR and average power output in the first 60 seconds. Further analyses of pacing across the two conditions showed large differences—again in favour of the high identity entrepreneurship condition—in gains in average power output from baseline in the early stages of Trial 2. This, to our knowledge, is the first demonstration of the positive effect that leaders’ identity entrepreneurship can have on group members’ effort and performance. As a result, our findings have several important theoretical and practical implications.

First, findings complement, and extend, previous research that points to the benefits of leaders’ identity entrepreneurship. Previous research in political and organizational contexts has shown that this is positively related to leaders’ capacity to engender both followers’ support [27] and group members’ engagement [25, 26]; of particular relevance to the present study, qualitative research in elite sport has pointed to the potential for acts of identity entrepreneurship (e.g., using collective language and promoting team clothing) to contribute to team members’ success [47]. Our findings indicated a positive effect of identity entrepreneurship on both effort (in terms of maximum heart rate) and performance (in terms of average power output in the first 60 seconds, while there was no effect on overall average power output or time taken). It is notable that, in the high identity entrepreneurship condition, the largest performance gains were observed at the start of Trial 2—immediately after the leader delivered the manipulation (either the initial statement or a reinforcement). It is plausible that, although in the high identity entrepreneurship condition participants’ willingness to invest effort, and desire to perform well, may have been greater throughout Trial 2, exerting more effort, and therefore generating a high power output, at the beginning of Trial 2 caused early fatigue and prevented them from improving their overall performance to a greater extent. That is, participants in the high identity entrepreneurship condition may have exceeded their lactate threshold in the early stages of Trial 2, with the elevated concentration of lactate in their blood impairing their performance during the remainder of the trial (e.g., see [71–73]). In addition, it is also possible that the effect on group members’ effort and performance ‘wears off’ in the absence of continued interaction and reinforcement. This suggests that further tests of relationships between leaders’ identity entrepreneurship and group members’ effort and performance—for example, using physical tasks of different durations (and measuring blood lactate concentration as a further indicator of effort) or cognitive tasks where the effort required to perform well is mental rather than physical—are needed to establish the factors underpinning the effects observed here.

The present findings also extend growing evidence for the benefits of identity leadership in sporting settings [6, 47]. In particular, by manipulating (one facet of) identity leadership, our findings build on Fransen et al.’s [6] experimental study that identified a positive association between leaders’ engagement in identity leadership and group members’ performance. Rather than examining identity leadership as a mediator, however, here we examined this as an independent variable that can shed light on causality. Moreover, our findings provide evidence of a mechanism through which leaders’ engagement in identity leadership impacts group members’ performance—namely increased effort. Indeed, given (1) the stripped-down nature of our task—such that the impact of several additional factors (e.g., technique, concentration, co-ordination between team members) that may, in other contexts, influence performance were removed (or at least minimized), and (2) the standardized conditions in which participants undertook the task (i.e., controlling for the potential influence of environmental factors such as ambient temperature), our findings give some confidence that this is likely to be a key determinant of the differential performance effects we observed (particularly in the early stages of our trials) in the high and low identity entrepreneurship conditions.

More generally, our findings align with a body of evidence that indicates the potential for group processes to influence members’ effort. Of particular note, experimental research has pointed to the role that increases in group cohesion can play in both (a) reducing social loafing effects (i.e., reductions in group members’ motivation and effort when individuals work collectively rather than individually; see [30, 31, 62]) and, on the other hand, (b) negatively influencing group members’ effort [29], depending on the content of the group norm that is established. Our findings extend previous research in this area by pointing to (1) the role that leaders can play in influencing group processes and thus group members’ effort, (2) leaders’ potential to exert instantaneous effects (i.e., as an adjunct to evidence for the benefits of extended team building programmes; see [30]), and (3) the value of targeting an additional group construct in attempts to improve members’ efforts (i.e., social identification). That is, although identification and cohesion are closely related (e.g., see [74]), they are nevertheless distinct constructs. Group cohesion refers to the extent to which, during both task and social interactions, members feel attracted to, and integrated into, the group [75]. Group identification refers to the strength of individuals’ social psychological connection to the group, and emerges following a psychological shift in the way people define themselves from a personal to a social identity [32, 33, 76]. Thus, although some elements of the multidimensional construct of cohesion may have been enhanced through our high identity entrepreneurship manipulation, key elements of cohesion (e.g., promoting positive social interactions between group members; see [75]) were not the target of our manipulation. Instead, we aimed to provide leaders with the tools to help create a shared sense of *identity* among members (i.e., a sense that they are part of the same group and an understanding of what it meant to be a member of that group—in this case to try hard and aim to win the perceived competition). Importantly, our manipulation check indicated that confederate leaders were judged to have achieved this more successfully in the high identity entrepreneurship condition than in the low identity entrepreneurship condition.

Our findings have a number of implications for sport and exercise leaders. Not least, this is because team members’ effort and performance are key outcomes that leaders seek to increase. Indeed, even though improving group members’ performance will not always be a key objective for these leaders (e.g., fitness class instructors), enhancing group members’ effort is still likely to be. For example, fitness class members are likely to derive greater physiological benefits (e.g., greater weight loss) by exerting more effort during sessions and thereby enhancing the proportion of time they spend exercising within ‘fat burning’ HR zones [77]. Contradicting traditional approaches that promote the great ‘I’ in leadership (e.g., the great man approach; [78]), our findings suggest that, in order to promote these key outcomes, leaders should attend to the ‘we-ness’ of leadership [27]. That is, they should focus on presenting themselves as part of (rather than above) the group, and behave in ways that create a shared sense of identity among group members [18]. Speaking directly to the way in which leaders on the ground can accomplish this, our findings support Steffens and Haslam’s [27] assertion that we-referencing language is one simple and powerful tool that leaders can easily deploy. More generally, for those concerned with training leaders (e.g., in sport and exercise settings), our findings suggest that greater attention to developing leaders’ capacity to create (and manage) group identities is needed. To this end, administrators of leadership training programmes may look to incorporate aspects of the recently developed 5R programme, which, among other benefits, has been shown to improve organizational leaders’ capacity to engage in identity leadership [79], and group members’ perceptions of their sporting leaders’ engagement in identity leadership [80].

### Limitations and future research

Against the backdrop of this study’s strengths, which included its novelty and research design (which allowed causality to be tested), some limitations and avenues for future research should be noted. First, although the controlled laboratory setting in which our experiment was conducted could be considered an additional strength, this setting—and the artificial situation we created within it—differs from real-world sport and exercise settings in several respects. Perhaps most notably, in sport and exercise settings, groups often have both formal leaders (who have commonly been elected by the group or appointed based on their expertise; e.g., captains, coaches, exercise class instructors) and informal leaders (who emerge over time and fulfil various leadership roles; [81, 82]). In the present study, formal leaders were not appointed in this way, while informal leaders were given little time to emerge and their impact was not assessed. To confirm the ecological validity of our findings, future research might therefore explore the impact of real-world leaders (both formal and informal) engaging in identity entrepreneurship on group members’ effort and performance. Nevertheless, the influence of our confederate leaders, who, as far as participants were aware, had been appointed arbitrarily and were no more qualified for the role than themselves or any other group member, has positive implications for the potential impact of real-world leaders whose status within the group may afford them greater influence. Indeed, given that, in the present study, performance effects in the experimental group were strongest at the beginning of time trials—immediately after the manipulation—it is possible that real world leaders who are able to demonstrate their identity entrepreneurship throughout exercise bouts (e.g., in fitness classes) may exert an even greater impact on group members’ behaviors.

We hope that the present findings lay the foundation for, and help stimulate, future efforts to examine these possibilities. Indeed, it is important to reiterate that, from a social identity perspective, effective leadership is not solely contingent on leaders’ identity entrepreneurship (i.e., their capacity to *create* a shared sense of identity among group members) but also on their capacity to *represent* and *advance* that identity, and help *embed* it in reality. As such, there is considerable scope for future research to explore both the distinct and combined effects of real-world sport and exercise leaders engaging in the four facets of identity leadership, particularly in light of recent evidence for context-specific differences in the relative importance of sporting leaders engaging in each of these [83].

We also note potential limitations associated with our manipulations. Ensuring that participants perceived these to be credible and reflective of what a ‘real’ leader might say was paramount and, for this reason, we were unable to use manipulations that precisely mirrored each other in all aspects aside from their identity-related content. Indeed, with believability again in mind, the messages designed to reinforce the manipulation were delivered in the context of congratulatory or encouraging messages. We acknowledge that this approach may have resulted in unintended additional benefits beyond those that arose from participants perceiving the leader to have created a stronger sense of identity among group members. Thus, although the results of our manipulation check indicated differences between the two conditions regarding the extent to which leaders were perceived to engage in identity entrepreneurship, we are unable to rule out the possibility that other factors (besides identity) had an additional impact on participants’ effort and performance. Researchers may therefore consider including additional measures (e.g., of perceived task importance and desire to outperform other participants) in future research so that these potential confounds can be controlled for statistically.

Our final sample size (following the removal of four groups from our analyses) could be considered a further limitation. Although, as noted above, our sample was able to detect effects of smaller sizes than those observed by Rees et al. [50] and Hoigaard et al. [45], larger cell sizes would be advisable in future research. Researchers seeking to conduct further experimental research to build on the present study should also carefully consider group size. Similar findings to those reported in the Results section were observed when participants who conducted Trial 2 in groups of three were included in our analyses. That is, with the exception of average HR (which became significant), the significance (or otherwise) of the differences between the two conditions’ gain scores were unchanged. Nevertheless, given that real-world exercise groups, sports teams, and groups and teams in other domains often consist of well over five people, it is particularly important that researchers test—both experimentally and through interventions—the impact of identity entrepreneurship (and identity leadership more broadly) on group members’ effort and performance in larger groups. Along similar lines, given that many real-world groups and teams are mixed-gender (and that the gender of leaders and group members will often differ), it is also important that future research examines the impact of identity entrepreneurship on group members’ effort and performance in groups with all leader and group member gender combinations. Indeed, although no significant condition by gender interactions were observed in the present study, our sample size precluded comparisons of the relative impact of our manipulation in male and female groups and this might therefore also be a focus for future research.

## Conclusion

The present findings support claims that leaders are likely to be more effective the more they are able to create a sense of shared social identity within the group they are attempting to lead [26, 27, 36]. More specifically, our findings provide the first causal evidence that, by creating this sense of shared ingroup identity, leaders can foster improvements in (or at least help maintain) group members’ effort and performance. In short, it appears that one key way in which leaders can motivate group members to try harder, and thereby help deliver better group outcomes, is by cultivating a strong sense of ‘us’ within the teams they lead. Indeed, if, as Fernando Torres (the former Spain international—World Cup, European Championships, and Champions League winner) observed, “we win as a team and every individual is better if we are part of the team”, then one might consider the first task of leadership to be building social identity.

## Supporting information

S1 Appendix Details of multilevel analyses examining the impact of the experimental manipulation on indicators of effort and performance. (DOCX)
