# Supplementary material for: Leaders’ creation of shared identity impacts group members’ effort and performance: Evidence from an exercise task
Source: PLoS One. 2019 Jul 11;14(7):e0218984. doi: 10.1371/journal.pone.0218984 (PMC6622477; doi:10.1371/journal.pone.0218984)
Supplement: S1 Appendix — (DOCX) [file pone.0218984.s001.docx]

**S1 Appendix**

As noted in the manuscript, multilevel analyses accounting for the nested nature of our data were conducted and are reported here for descriptive purposes. Specifically, we analysed the impact of the manipulation on each indicator of effort and performance through multilevel modelling (in SPSS version 25) with maximum likelihood estimation. Results were similar to those reported in the main manuscript and were as follows (*p* values are one-tailed in line with our directional hypotheses and reporting in the manuscript): (1) average HR: *b* = 3.82, SE = 2.41, *p* = .065 (2) maximum HR: *b* = 3.69, SE = 1.89, *p* = .034, (3) time taken: *b* = -5.18, SE = 5.72, *p* = .19, (4) average power output: *b* = .270, SE = 5.30, *p* = .309, (5) average power output first 60 seconds: *b* = 25.94, SE = 11.07, *p* = .016.
